# The 'three Ps' of cancer survivorship care

**DOI:** 10.1186/1741-7015-9-14

**Published:** 2011-02-10

**Authors:** Patricia A Ganz

**Affiliations:** 1 UCLA Schools of Medicine and Public Health, Division of Cancer Prevention & Control Research, Jonsson Comprehensive Cancer Center, Los Angeles, CA, USA

## What is the definition of 'cancer survivor' and what is 'survivorship care'?

**Figure 1:**
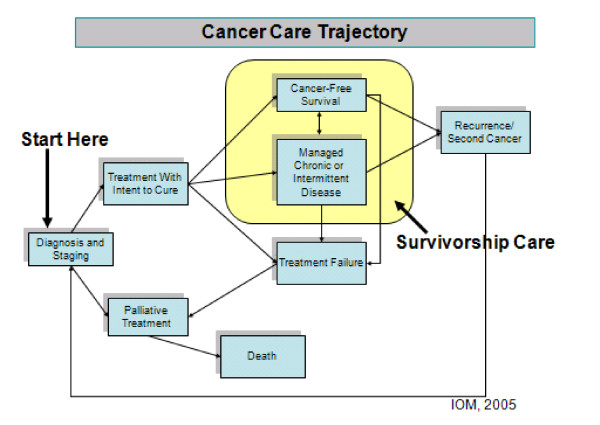
**Cancer care trajectory and survivorship care**. Figure adapted from the Institute of Medicine. Reprinted with permission from *From Cancer Patient to Cancer Survivor: Lost in Transition*, 2005, by the National Academy of Sciences, Courtesy of the National Academies Press, Washington, DC, USA.

With the growing numbers of individuals surviving long term and disease free after a cancer diagnosis, a group of individuals in the US came together in 1986 to establish the National Coalition for Cancer Survivorship (NCCS; http://www.canceradvocacy.org), and defined cancer survivors as individuals with cancer from the time of diagnosis and for the balance of life, and included in its definition family, friends and caregivers. This broad and all-inclusive definition reminds us that potentially anyone with a diagnosis of cancer can be a long-term survivor and that we must design therapies at the outset to maximize cure as well as minimize long-term side effects and other more serious effects of cancer treatments. In practical terms, however, we tend to focus on the post-treatment phase of the cancer experience, and that is where survivorship care really gets started (Figure 1).

## Why is survivorship care something that needs dealing with?

The reason for the focus on survivorship care is that it has been largely neglected. The medical care system primarily focuses on curing the cancer and not on how to manage the after effects. Patients are often left wondering about how often they need to be seen after treatment ends and what follow-up tests need to be performed. In addition, they are often troubled by persistent symptoms and side effects of treatment that the medical care system seldom attends to, and these problems may cause ongoing suffering and burden. Finally, cancer survivors often neglect other aspects of their health and may remain at risk for second cancers and other serious chronic diseases (for example, heart disease, diabetes).

## What are the 'three Ps'?

As I began lecturing about survivorship care I wanted to come up with something catchy to help people remember the important components of post-treatment survivorship care. Conceptually, the care delivery falls into three major domains: **Palliation **of ongoing symptoms; **Prevention **of late effects of cancer treatment or second cancers; and Health **Promotion **to maximize future wellness. For example, the cancer may be gone, but patients still can suffer from pain, fatigue or depression, for which palliative care approaches become central. Prevention of the late effects means monitoring and preventing common late effects such as osteoporosis, screening for second cancers (for example, skin exams, breast cancer after chest radiation). Health promotion is often overlooked because everyone is concentrating on monitoring for cancer recurrence, when in fact many adults are at risk for and will die from cardiovascular disease, stroke or diabetes complications. Making sure that all three Ps are addressed is an important aspect of survivorship care.

## What led to the development of these concepts?

I was a member of the Institute of Medicine (IOM) committee that prepared a report on the challenges associated with addressing the needs of post-treatment cancer survivors (see Hewitt *et al*, 2006). This report documented the growing number of cancer survivors living after treatment ends and how they were 'lost in transition' with little guidance or coordination in their health care, and their unmet health care needs. The report clearly states that there is a need to acknowledge this phase of the cancer care trajectory, to do more research to better understand how care should be provided, and to focus on improvement in the coordination and quality of care through use of a treatment summary and survivorship care plan.

## What is the IOM?

The IOM is part of the US National Academy of Sciences and is responsible for providing independent unbiased advice on issues related to biomedical science, medicine, and health, and its mission to serve as adviser to the nation to improve health. It works outside the framework of the US federal government to provide independent guidance and analysis and relies on a volunteer workforce of scientists and other experts, operating under a rigorous, formal peer-review system (see http://www.IOM.edu).

## How well are these strategies being taken up in the US?

In the 5 years that have elapsed since the IOM report on adult cancer survivors, there has been increasing awareness of the growing number of cancer survivors in the US (now numbering about 12 million, or 4% of the population), as well as increasing efforts at major cancer centers to develop programs that will improve the post-treatment management of cancer patients. However, widespread adoption of some of the key strategies and recommendations has been limited, with the exception of many of the US National Cancer Institute comprehensive cancer centers. This is mostly because about 80% of cancer care in the US occurs in community settings and not in major cancer centers. Small groups of oncology practitioners are the norm, and the surgeons and radiation oncologists often do not practice in the same setting with the medical oncologists. Thus, the acute treatment and post-treatment care is often fragmented, and usually does not include the general practitioner. This makes it difficult to coordinate post-treatment care and to ensure that the three Ps are delivered when patients are in follow-up.

## How about other parts of the world?

I think that the needs of cancer survivors are just beginning to get attention in other parts of the world. The UK, Australia, Canada and parts of the EU now have some of these same issues on their radar and are developing relevant models of care.

## Are there any special cases?

For childhood cancer survivors, the situation is a bit more organized. They are fewer in number and are almost all treated at specialized cancer centers across the world. These centers often follow-up children who were treated many years ago long into adulthood (for example, for 30-40 years). These children often experience very serious side effects from treatment (for example, short stature, endocrine deficiencies, second cancers, premature cardiac disease) and as a result they are more closely monitored. In the US, guidelines have been developed for their care (see http://www.survivorshipguidelines.org/) by the Children's Oncology Group (COG), and many follow-up clinics exist that are using these guidelines which focus on the three Ps.

## Where can I find out more?

Hewitt M, Greenfield S, Stovall E (Eds): *From Cancer Patient to Cancer Survivor: Lost in Transition*. Washington, DC, USA: The National Academies Press; 2006.

Armstrong GT, Liu Q, Yasui Y, Neglia JP, Leisenring W, Robison LL, Mertens AC: **Late mortality among 5-year survivors of childhood cancer: a summary from the Childhood Cancer Survivor Study**. *J Clin Oncol *2009, **27**:2328-2338.

Ganz PA, Casillas J, Hahn EE: **Ensuring quality care for cancer survivors: implementing the survivorship care plan**. *Semin Oncol Nurs *2008, **24**:208-217.

Ganz PA: **A teachable moment for oncologists: cancer survivors, 10 million strong and growing! ***J Clin Oncol *2005, **23**:5458-5460.

